# MRI analysis of carpal tunnel syndrome in hemodialysis patients versus non-hemodialysis patients: a multicenter case-control study

**DOI:** 10.1186/s13018-019-1114-0

**Published:** 2019-03-28

**Authors:** Koji Fujita, Kenji Kimori, Akimoto Nimura, Atsushi Okawa, Yoshikazu Ikuta

**Affiliations:** 1 Department of Orthopaedic and Spinal Surgery, Graduate School of Medical and Dental Sciences, Tokyo Medical and Dental University, 1-5-45, Yushima, Bunkyo-ku, Tokyo, 113-8519 Japan; 2 Department of Orthopedic Surgery, Tsuchiya General Hospital, Hiroshima, Japan; 3 Department of Functional Joint Anatomy, Graduate School of Medical and Dental Sciences, Tokyo Medical and Dental University, Tokyo, Japan

**Keywords:** Hemodialysis, Carpal tunnel syndrome, MRI scan, Cross-sectional area

## Abstract

**Background:**

Carpal tunnel syndrome (CTS) is common among patients receiving hemodialysis and deeply influences their daily life. Amyloid deposits are considered the main reason for median nerve compression, but its prevalence is unclear. Therefore, to determine the main region of amyloid deposition inside the carpal tunnel, we measured the cross-sectional area (CSA) of each component of the carpal tunnel in preoperative magnetic resonance imaging (MRI).

**Methods:**

Thirty-five hemodialysis patients (HD group) and age- and sex-matched 35 non-hemodialysis patients (non-HD group), who underwent the first surgery for CTS in registered hospitals from 2005 to 2015, were retrospectively enrolled. CTS was diagnosed from clinical and electromyographic (EMG) findings. The CSA of carpal tunnel, each of the flexor tendons, and the median nerve at the level of the hook of hamate were measured in T1-weighted axial images in preoperative MRI, by using Synapse OP-A software. Statistical analysis was performed using the Student’s *t* test and Pearson’s chi-squared test.

**Results:**

The mean age of the HD group was 65.9 years and the dialysis duration was 21.9 (11–35) years. The mean age of the non-HD group was 65.3 years. The CSA of carpal tunnel (*p* = 0.006), flexor tendon (*p* = 0.03), and flexor digitorum profundus (FDP) tendon (*p* = 0.04) were bigger in the HD group. However, the median nerve, the flexor digitorum superficialis (FDS) tendon, and the flexor pollicis longus tendon (FPL) were not significantly different between the two groups. The dialysis duration or age at surgery did not show any strong correlation to each CSA.

**Conclusions:**

We confirmed that hemodialysis caused expansion of the carpal tunnel due to amyloid deposition as previously described. Hemodialysis also caused expansion of the CSA of the flexor tendon, especially the FDP, possibly because of amyloid deposition inside the tendon. Furthermore, the duration of dialysis or age did not correlate with any CSA, which could be due to the good progress of the beta 2-microglobulin removal technique. Based on our results, FDS excision could be considered in case severe deposition of amyloid in FDP is observed during surgery.

## Background

Carpal tunnel syndrome (CTS) is common among patients receiving hemodialysis and deeply influences their daily life [1–3]. Amyloid deposits are considered as the main reason for median nerve compression [4–6], and long-term HD therapy was found to cause a higher occurrence of CTS. Carpal tunnel release for HD patients showed reasonable clinical results primarily [7, 8], but a higher recurrence rate was observed in contrast to that in non-HD patients in CTS [6, 9, 10]. The type of HD or the side of arteriovenous fistula was reported as a risk factor for developing CTS [2, 7, 11]. However, the detailed mechanism of amyloid deposition, such as the location of deposit and its mechanism, is not clearly understood.

In this study, to identify the main region of amyloid deposition inside the carpal tunnel and examine its mechanism, we measured the cross-sectional area (CSA) of each composition of the carpal tunnel in preoperative magnetic resonance imaging (MRI) in HD patients who would have carpal tunnel release surgery with a duration of hemodialysis of more than 10 years, in comparison to age- and sex-matched non-HD patients who would have carpal tunnel release surgery.

## Methods

### Patients

The institutional review board approved this study. We conducted a multicenter case-control study based on retrospective chart and image review of the patients who underwent primary carpal tunnel release surgery in two registered hospitals. Thirty-five HD patients (HD group) who underwent surgery from 2005 to 2016, with more than 10 years of HD, and sex- and age-matched 35 control non-HD patients (non-HD group) who underwent surgery in the same period, with a diagnosis of idiopathic CTS, were included. HD was performed three times a week by using the forearm arteriovenous fistula in all patients of the HD group. We collected data about the age, sex, affected side, the side of arteriovenous fistula when the surgery was performed, duration of HD, and the CSA of each component inside the carpal tunnel in preoperative MRI, details of which are described below.

CTS was diagnosed by well-trained hand surgeons, based on symptoms and physical and electromyographic findings [12]. We excluded the patients who had surgeries on both sides or revision cases.

**Fig. 1:**
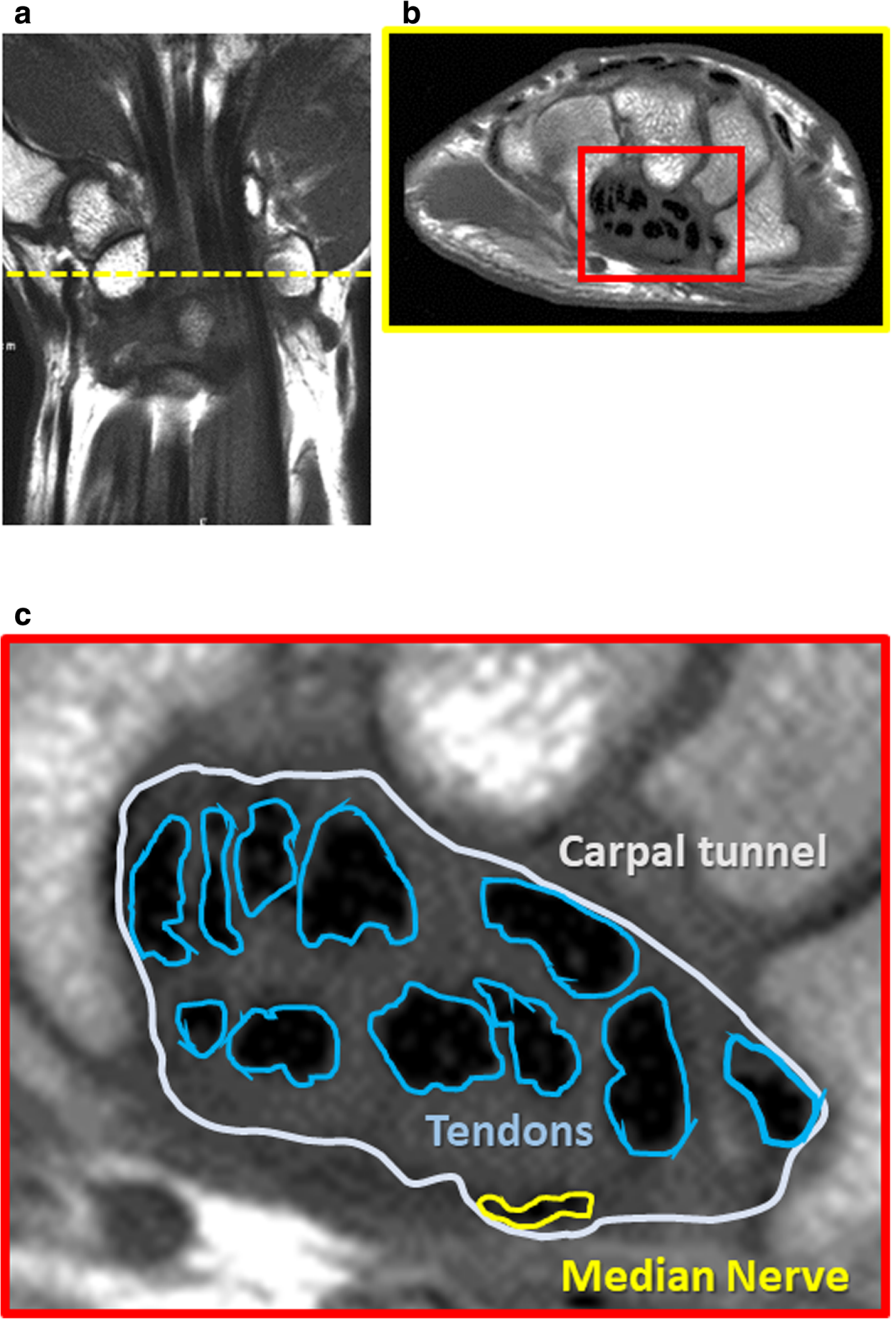
The method of measuring the CSA. In T1-weighted axial image of MRI, at the level of the hook of hamate (**a**) with the axis of which was vertical to the line from the third web space to the ulnar edge of the palmaris longus tendon (**b**), the CSA of carpal tunnel, each flexor tendon, and median nerve were calculated (**c**)

We routinely performed preoperative MRI scan (Gyroscan Intera-Achieva 1 .5T Master R1.5, Philips, Best, NL) for all the carpal tunnel patients right before the surgery and measured the CSA of the carpal tunnel, the flexor tendons, and the median nerve. The CSA was measured using Synapse OP-A software at the level of the hook of hamate in T1-weighted axial image, the axis of which was vertical to the line from the third web space to the ulnar edge of the palmaris longus tendon, in preoperative MRI. (Fig. 1).

### Statistical analysis

The Student’s *t* test was used to compare the CSA in each group. A *p* value < 0.05 was considered as statistically significant. The Pearson correlation coefficient was used to analyze the correlation between the duration of hemodialysis or age and CSA.

## Results

In our study, 22 females and 13 males were included in each group. There was no significant difference in age (*p* = 0.92). Twelve patients of the HD group and 25 of the non-HD group were affected in their right hand. In the HD group, 12 patients had arteriovenous fistulas in their right forearms, and 21 cases were affected on the arteriovenous fistula side (Table 1).

**Table 1:** Data of patients’ characteristics

–	Control (*n* = 35)	HD (*n* = 35)	*p* value
Age* (years)	65.9 (6.7)	65.3 (10.5)	0.78
Female/male^†^	22/13	22/13	
Duration of HD* (years)		21.9 (6.8)	
Affected side (right/left)^†^	25/10	12/33	
Arteriovenous fistula side (right/left)^†^		14/21	
Affected side = arteriovenous fistula side^†^		21	

Statistical significance was determined with Student’s *t*-test

*Abbreviations*: *HD* hemodialysis

*Data are presented as the median (standard error)

^†^Data are presented as the number of the patients.

The CSA of the carpal tunnel was significantly bigger in the HD group than in the non-HD group (*p* = 0.006). The CSA of total the flexor digitorum superficialis (FDS) and the flexor pollicis longus (FPL) were not significantly different (*p* = 0.14, 0.19, respectively), but that of the total flexor digitorum profundus (FDP) was significantly bigger in the HD group (*p* = 0.04). The CSA of total flexor tendons was significantly bigger in the HD group. No difference was observed in the CSA of the median nerve (*p* = 0.72). The CSA of the third space, which was indicated by the CSA of the flexor tendon and median nerve subtracted from that of the carpal tunnel, was bigger in the HD group but not significantly (*p* = 0.06) (Table 2).

**Table 2:** Cross-sectional area of each component inside carpal tunnel

–	Control	HD	*p* value
Carpal tunnel (cm^2^)	174.1 (3.7)	194.3 (6.1)	0.006
Total of FDS (cm^2^)	39.0 (1.5)	42.7 (2.0)	0.14
Total of FDP (cm^2^)	48.7 (1.5)	54.5 (2.4)	0.04
FPL (cm^2^)	9.0 (0.5)	10.1 (0.7)	0.19
Total of flexor tendons (cm^2^)	96.7 (2.6)	107.3 (4.1)	0.03
Median nerve (cm^2^)	5.4 (0.6)	5.1 (0.5)	0.72

Data are presented as the median (standard error). Statistical significance was determined with Student’s *t* test and *p* < 0.05 was considered as significant

*Abbreviations*: *FDS* flexor digitorum superficialis, *FDP* flexor digitorum profundus, *FPL* flexor pollicis longus

**Fig. 2:**
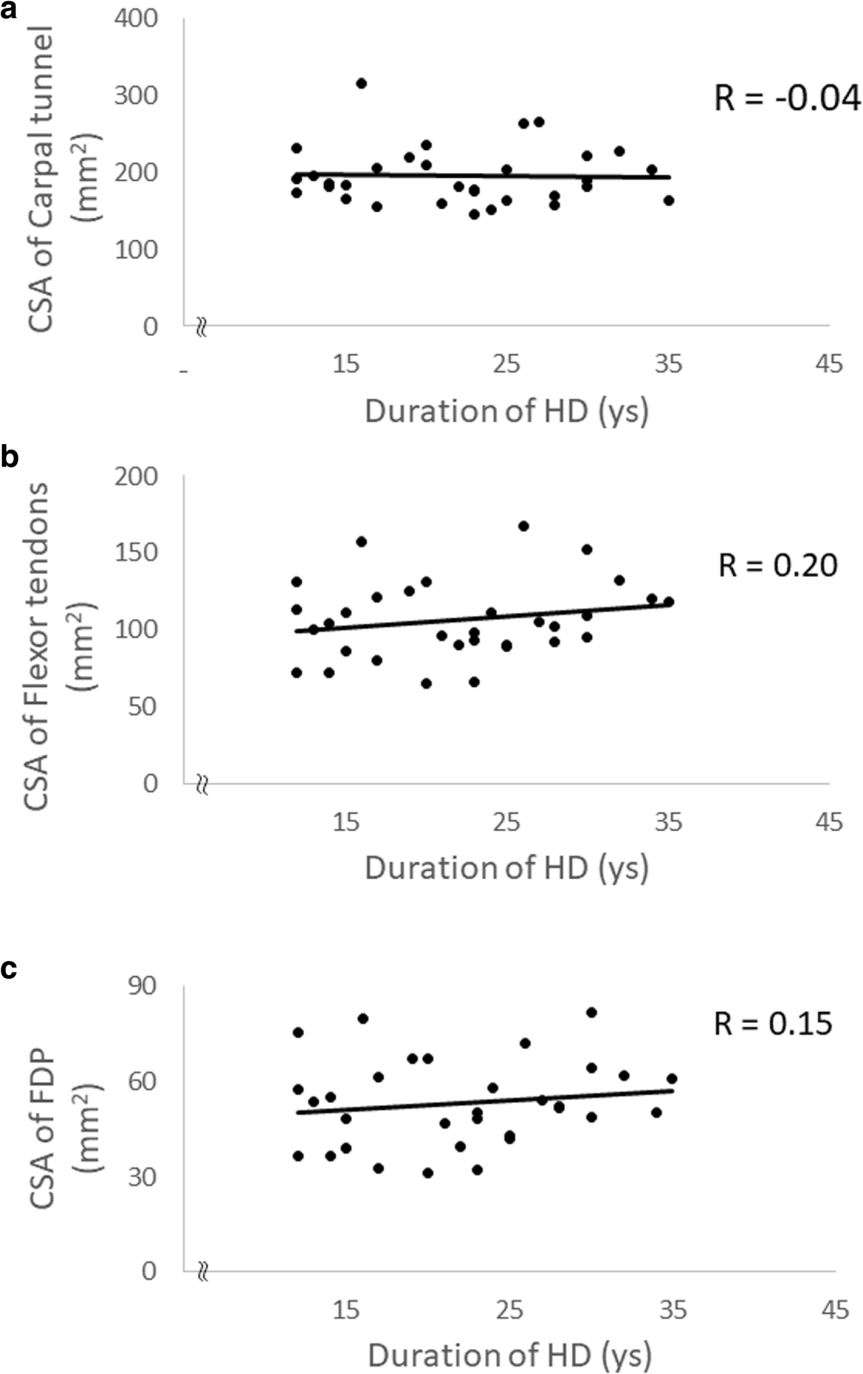
Scatter plots showing the correlation between the duration of HD and each component in the carpal tunnel. Neither of the CSA of carpal tunnel ((**a**), *R* = − 0.04), flexor tendons ((**b**), *R* = 0.20), or FDP ((**c**), *R* = 0.15)) showed a strong correlation

**Fig. 3:**
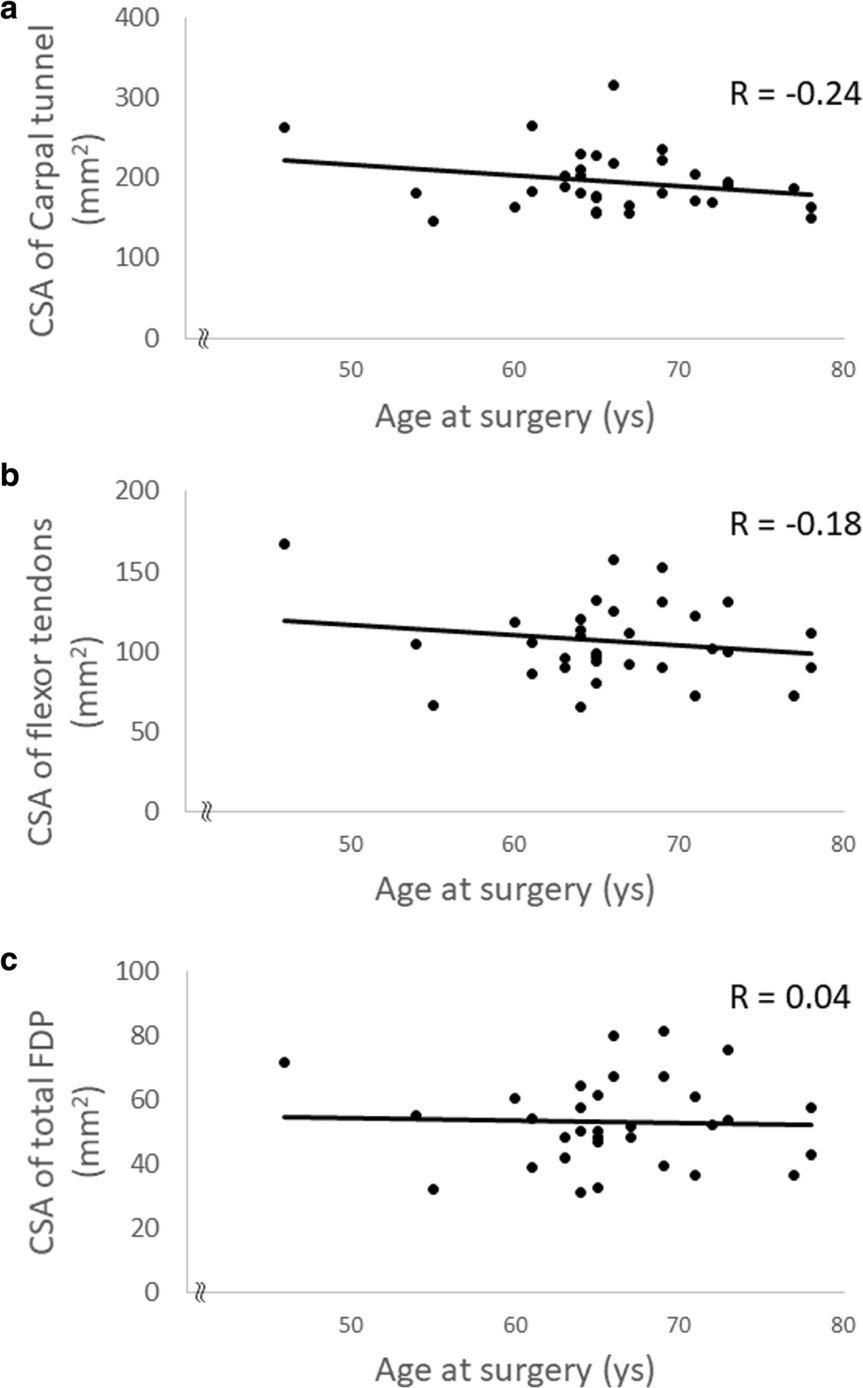
Scatter plots showing the correlation between age at surgery and each component in the carpal tunnel. Neither of the CSA of carpal tunnel ((**a**), *R* = − 0.24), flexor tendons ((**b**), *R* = − 0.18), or FDP ((**c**), *R* = 0.04) showed a strong correlation

In the HD group, a strong correlation was not observed between the CSA of each component and the duration of HD (Fig. 2). Also, the CSA of each component and the patients’ age did not show any strong correlation (Fig. 3).

## Discussion

In this multicenter case-control study, we measured the CSA of each component inside the carpal tunnel of HD patients with CTS in comparison to age- and sex-matched non-HD patients with CTS. We found that the CSA of FDP was significantly bigger in the HD than the non-HD group, although the other components did not show statistical differences. In the HD group, the age of patients or the duration of HD did not show a strong correlation with each CSA.

Although the occurrence of CTS in HD patients is much higher than that in non-HD patients [6, 13], the mechanism which increases the pressure of the carpal tunnel is not well understood. Previous studies have shown mixed results with respect to the etiology. The thickening of the palmar radiocarpal ligament due to amyloid deposition was reported as one of the reasons which caused increased pressure inside the carpal tunnel same as idiopathic CTS [14]. Amyloid deposition inside the carpal tunnel including components is reported as another cause of HD-related CTS, as the CSA of the carpal tunnel or the median nerve increases gradually due to amyloid deposition with the passage of HD duration [15, 16].

This study showed the increased CSA of FDP in HD patients. Amyloid deposition normally occurs in the tissues with good blood flow, such as the artery, synovial tissue, and bone [17, 18]. The tendon is considered as a low blood flow tissue [19, 20], but in this study, the increased CSA revealed the deposition of amyloid in the tendons. A possible explanation of this symptom is that the FDP lies in a deeper area of the carpal tunnel with better blood flow compared to the FDS and the developed synovial tissues with good blood flow enveloped the FDP and provided amyloid deposit into and around FDP. During the surgery, we observed more amyloid deposits and synovium around the FDP in HD patients than in non-HD patients, which could be the reason of the high recurrence rate.

In one patient with HD-related CTS, who was not included in this analysis because it was a revision case, we dissected the accessory tendon of the FDP to reduce the volume inside the carpal tunnel, and then examined the accessory tendon histologically. Amyloid deposition was confirmed not only around but inside the tendon. This result showed that amyloid tends to deposit around and inside the FDP, and both preoperative examination by MRI or ultrasonography and intraoperative observation of FDP might be important options when performing the surgery. This may also suggest that when a massive amyloid deposition in the FDP was observed, excising the accessory tendon or ulnar half-slip of FDS is a feasible option. The FDS excision reduces the gliding resistance and volume inside the carpal tunnel [21].

The previous cohort study showed the increased occurrence of CTS in long-term HD patients [22]. However, in this study, we found no correlation between the duration of HD and the CSA of carpal tunnel or FDP. This discrepancy may be caused by the improvement of dialysis technology to remove beta-2 microglobulin from blood flow [23]. In Japan, the dialysis patient population has been growing every year, and it was over 320,000 by the end of 2015 [24]. Along with the expansion of demand, an impressive advancement of dialyzers for hemodialysis has been made, and the clearance of beta-2 microglobulin has improved year by year [25, 26]. Over 80% clearance of beta-2 microglobulin is considered to be effective to reduce the risk of CTS occurrence, but the rate of clearance depends on the patients’ status and may vary in each patient [15]. This may be the reason we could not see the correlation between the duration of HD and the CSA.

Our results did not show the relationship between the side of arteriovenous fistula and the affected side. This result may be explained by amyloid deposits being carried by blood flow. Moreover, we need to pay attention to the amyloid deposition in the other joints and organs when we examine the HD patients with CTS.

Several limitations of this study should be acknowledged. First, we could not assess the risk factors of surgery for CTS. If we had chosen the control group from patients without CTS surgery with more than 10 years of HD, we could have assessed the risk of CTS occurrence. However, to perform MRI scans on the patients without CTS has low feasibility. Second, the cause of hemodialysis installation like renal diseases, diabetes, or autoimmune disease could be a confounding factor for the CSA of carpal tunnel, but we could not access the information in this study. Third, we did not use ultrasonography in this study. Ultrasonography can assess the contents of the carpal tunnel in real time and the blood flow of the synovium; thus, we would like to use it in our next series.

## Conclusions

We compared the CSA of the carpal tunnels of HD patients to that of non-HD patients and found that HD caused amyloid deposition in and around the FDP tendons and significant expansion of the carpal tunnel. Based on our results, FDS excision could be considered in cases where severe deposition of amyloid in FDP is observed during surgery. Further examination of the risk factors of amyloid deposition and CTS surgery on HD patients is needed.

## Abbreviations

CSA: Cross-sectional area
CTS: Carpal tunnel syndrome
FDP: Flexor digitorum profundus
FDS: Flexor digitorum superficialis
FPL: Flexor pollicis longus
HD: Hemodialysis
MRI: Magnetic resonance imaging
